# A systematic review of brief respiratory, embodiment, cognitive, and mindfulness interventions to reduce state anxiety

**DOI:** 10.3389/fpsyg.2024.1412928

**Published:** 2024-06-12

**Authors:** Phoebe Chin, Faye Gorman, Fraser Beck, Bruce R. Russell, Klaas E. Stephan, Olivia K. Harrison

**Affiliations:** ^1^Department of Psychology, University of Otago, Dunedin, New Zealand; ^2^Ngāpuhi, New Zealand; ^3^Ngāti Kahu, New Zealand; ^4^Ngāi Tahu, New Zealand; ^5^Optimal Health Model Ltd., Glenorchy, New Zealand; ^6^School of Pharmacy, University of Otago, Dunedin, New Zealand; ^7^Translational Neuromodeling Unit, Institute for Biomedical Engineering, University of Zurich & ETH Zurich, Zurich, Switzerland

**Keywords:** anxiety, brief intervention, breathing, relaxation, cognition, mindfulness, interoception

## Abstract

**Introduction:**

Anxiety is one of the most prevalent mental health conditions worldwide, and psychotherapeutic techniques can be employed to help manage and mitigate symptoms. While the available therapies are numerous, key strategies often involve cognitive and/or embodiment techniques. Within body-centered methods, breathing-oriented approaches are particularly prevalent, using either attention towards or active control of breathing. As the perception of body states (i.e., interoception) is thought to be an integral component of emotion generation, these embodiment and breathing techniques may be key in addressing the miscommunication between the brain and body that is thought to exist with anxiety. Therefore, we conducted a systematic review and meta-analysis to assess the effects of acute administration of psychological interventions for state anxiety.

**Results:**

This systematic review was conducted in accordance with the PRISMA statement and registered prospectively in PROSPERO. A literature search for randomized controlled trials was conducted in PubMed, PsycINFO, and Scopus. We considered interventions that focused on cognitive, embodiment or breathing strategies, or a combination of these techniques. Twelve studies met our inclusion criteria, and study characteristics, quality and effect sizes were assessed. A single cognitive study was found to produce a moderate reduction in state anxiety, while moderate to large effects were found across studies assessing embodiment practices. In contrast, studies which utilized breathing-based interventions alone produced inconsistent results, with both attention towards and active control of breathing producing large to no effects depending on the technique employed. Finally, consistent moderate effects were found with combination techniques that involved passive attention (e.g., towards cognitions, body and/or breathing), with active combination techniques producing inconsistent results.

**Discussion:**

While study numbers are limited regarding brief interventions, cognitive and embodiment techniques are consistently helpful for reducing state anxiety, while breathing-based exercises need to consider the specific technique employed, and how successful this may be for each individual. Furthermore, combined practices such as mindfulness can also be successful, although care must be taken when introducing an active change to one or more elements.

**PROSPERO Systematic Review Registration Number:**

CRD42024507585 Available from: https://www.crd.york.ac.uk/prospero/display_record.php?ID=CRD42024507585.

## Introduction

Anxiety disorders are one of the most prevalent mental health disorders, with population-based research suggesting approximately 33.7% of individuals are impacted by at least one anxiety disorder throughout their life (Kessler et al., 2012). Subthreshold symptoms of anxiety are even more common than diagnostic-level anxiety disorders (Olfson et al., 1996; Haller et al., 2014). The prevalence of anxiety at both subthreshold and diagnostic levels has increased over time, particularly in younger populations (Goodwin et al., 2020; Yang et al., 2021). Several explanations have been proposed for these recent increases in anxiety, including the COVID-19 pandemic, and the marked rise in social media use (Santomauro et al., 2021; Braghieri et al., 2022; Peper, 2023). A cohort study conducted in New Zealand found that 53% of participants reported symptoms of anxiety, with 24% reporting moderate-to-severe anxiety (Gasteiger et al., 2021). Importantly, anxiety symptomology is broad and spans cognitive, affective, behavioral and physiological domains (Chand and Marwaha, 2023). Persistent symptoms can result in functional impairment, reduced quality of life, and an increased risk of progressing further to a clinical disorder if left unmanaged without any psychotherapeutic or pharmacotherapeutic input (Mendlowicz and Stein, 2000; Stein et al., 2005; Beard and Delgadillo, 2019).

Notably, individuals with anxiety have demonstrated impaired interoception (the perception of our body and inner physiological condition), which may contribute to deficits in recognizing physiological anxiety symptoms (Paulus and Stein, 2010; Seth, 2013). For example, individuals with moderate anxiety displayed lower respiratory-based interoceptive sensitivity and metacognitive bias during a breathing perception task, compared to those with low anxiety (Harrison et al., 2021), and decreased interoceptive awareness and cardiac sensitivity have also been associated with greater levels of depression and anxiety (Lackner and Fresco, 2016). This suggests a relationship between impaired interoceptive ability and the severity of anxiety symptoms. Therefore, improving interoception may be one of the mechanisms by which psychotherapeutic techniques act to produce the desired effects on anxiety.

Interventions for managing anxiety have been well-researched and cover a growing pool of psychological and pharmacological modalities. Within psychotherapeutic approaches for anxiety, traditional treatments were primarily cognitive and relaxation therapies. Techniques such as cognitive restructuring help individuals target and change distorted thoughts towards anxiety-inducing events (Goldfried et al., 1978). Common relaxation techniques include progressive muscle relaxation (PMR) and breathing exercises, which focus on changes to body and breathing modalities to reduce anxiety via a body-up mechanism. However, some studies have suggested that PMR and certain breathing exercises (i.e., hyperventilation breathing, deep breathing) can in fact intensify anxiety—especially with panic disorder (Meuret and Ritz, 2010; Curtiss et al., 2021; Maleki et al., 2022). In regard to breathing exercises, certain instructions (i.e., breathe shallower (inhaling less air) than usual) have been shown in some cases to perpetuate hyperventilation and exacerbate feelings of panic (Meuret et al., 2003; Conrad et al., 2007).

The most popular traditional psychotherapy utilized to address anxiety is cognitive-behavioral therapy (CBT), which includes a combination of both cognitive and relaxation exercises. This differs from alternative approaches such as interpersonal therapy (IPT) which places major emphasis on relieving symptoms by improving interpersonal functioning, rather than focusing on individual thoughts or behaviours (Markowitz et al., 2014). Several meta-analyses have found CBT to produce a large effect in treating anxiety disorders and reducing anxiety sensitivity (Smits et al., 2008; Cuijpers et al., 2016; Carpenter et al., 2018; Papola et al., 2023). CBT has also been shown to improve interoceptive abilities (Karanassios et al., 2021), although it is not yet known whether the cognitive or embodiment strategies may drive this change, nor whether one of these are an integral mediator for the reduction in anxiety.

Embodiment practices have also been utilized as a stand-alone technique for reducing anxiety. A recent meta-analysis provides strong support for this approach, where PMR—both alone and paired with other cues such as music, nature sounds and guided imagery—was found to be markedly effective in reducing stress, anxiety, and depression in adults (Muhammad Khir et al., 2024). Furthermore, specifically monitoring or manipulation of interoceptive cues through breathing attention or exercises may help treat diseases involving chronic elevated activity within the sympathetic nervous system, such as that associated with anxiety (Weng et al., 2021). Accordingly, breathing techniques have been shown to effectively reduce both physiological symptoms and anxiety levels (Jerath et al., 2015; Chen et al., 2017; Hopper et al., 2019), and techniques such as PMR have been hypothesized to act via interoceptive pathways (Cougle et al., 2020).

More recent third-wave approaches such as mindfulness and acceptance-based therapies emphasize focusing one’s *attention* towards thoughts and sensations rather than actively attempting to change such modalities (Hayes and Hofmann, 2021). For example, focusing attention towards one’s breathing without any attempt to actively manipulate breathing rate. Such mindfulness practices have been shown to both reduce anxiety symptoms significantly (Blanck et al., 2018) and increase interoceptive sensitivity compared to controls (De Lima-Araujo et al., 2022). Mindfulness-based stress reduction (MBSR), which involves focusing attention towards a *combination* of modalities (cognition, body, and breath), also improves anxiety symptom severity compared to control (Hofmann et al., 2010). Importantly, a recent meta-analysis by Papola et al. (2023) demonstrated that the use of behavioral and relaxation therapies, CBT, and third-wave interventions all significantly reduce symptoms in generalized anxiety disorder. Potentially mediating this effect, a review by Weng et al. (2021) described how interventions of bottom-up behavioral means (i.e., slowed breathing), and top-down psychological means (i.e., mindfulness) could alter interoceptive processing through physiological pathways. Improved interoceptive awareness has also been shown to facilitate the downregulation of negative affect when exposed to aversive stimuli (Füstös et al., 2013). Therefore, interventions involving the manipulation of one’s physiology as well as mindfulness-based strategies that target interoceptive pathways appear beneficial for managing and mitigating anxiety.

Clinical practice guidelines suggest therapies such as CBT to be conducted over 10 to 15 weekly sessions for anxiety-related disorders (Reddy et al., 2020). Hence, much of the psychotherapeutic literature has focused on the efficacy of the above approaches in a longitudinal manner typically observed in a clinical/therapeutic setting. Access to such long-term interventions is limited for many, with common barriers being the scarcity of mental health resources, stigma, lack of financial resources and logistical barriers such as difficulties with time commitments (Andrade et al., 2014; Waumans et al., 2022). One more accessible approach is to provide strategies that can be administered acutely, in a single session. Single-session approaches can help to reduce the risk of anxiety and anxiety symptoms (Schleider and Weisz, 2016, 2018; Bertuzzi et al., 2021), and immediate positive responses from an acute intervention may encourage individuals to continue to independently practice the relevant technique on a regular basis. However, the most beneficial approach (i.e., behavioural/relaxation, cognitive, third-wave) for mitigating state anxiety within a single-session is not yet clear.

Considering the high prevalence of the population experiencing anxiety symptoms, it is imperative to identify the most effective psychotherapeutic modalities for acute reductions in state anxiety. For this novel systematic review and meta-analysis, we aimed to determine which strategies are most impactful for reducing state anxiety. We considered the different aspects of traditional CBT and modern third-wave mindfulness/acceptance-based therapies (as two of the main psychotherapeutic interventions), including cognitive-, embodiment-, breathing-based strategies separately and in combination, to assess which individual components may be most efficacious to use in a single session.

## Material and methods

This review was guided by the Preferred Reporting Items for Systematic Reviews and Meta-analyses (PRISMA) 2020 statement which included a 27-item checklist. The review was prospectively registered in PROSPERO (CRD42024507585).

### Search strategy and selection process

A literature search was conducted of PubMed (National Library of Medicine), PsycINFO (Ovid), and Scopus (Elsevier) in October 2023. Databases were searched using the search strategy: (Brief OR Acute OR Immediate) AND (Breath* OR Respir* OR Relax* OR “Progressive Muscle Relaxation”) AND (Practice OR Intervention) AND (Anxiety OR “Anxiety Sensitivity” OR “Anxiety Rating” OR “Anxiety Measure”). Search results were limited to articles published in English using human participants. The references of the included articles were additionally screened for other relevant studies. Inclusion criteria were: (1) adults aged over 18 with no medical diagnoses; (2) experimental trials investigating the effects of an acute administration of cognitive, respiratory, or embodiment techniques (or a combination); (3) contained a measure of state anxiety as an outcome; and (4) randomized clinical trials.

**Figure 1:**
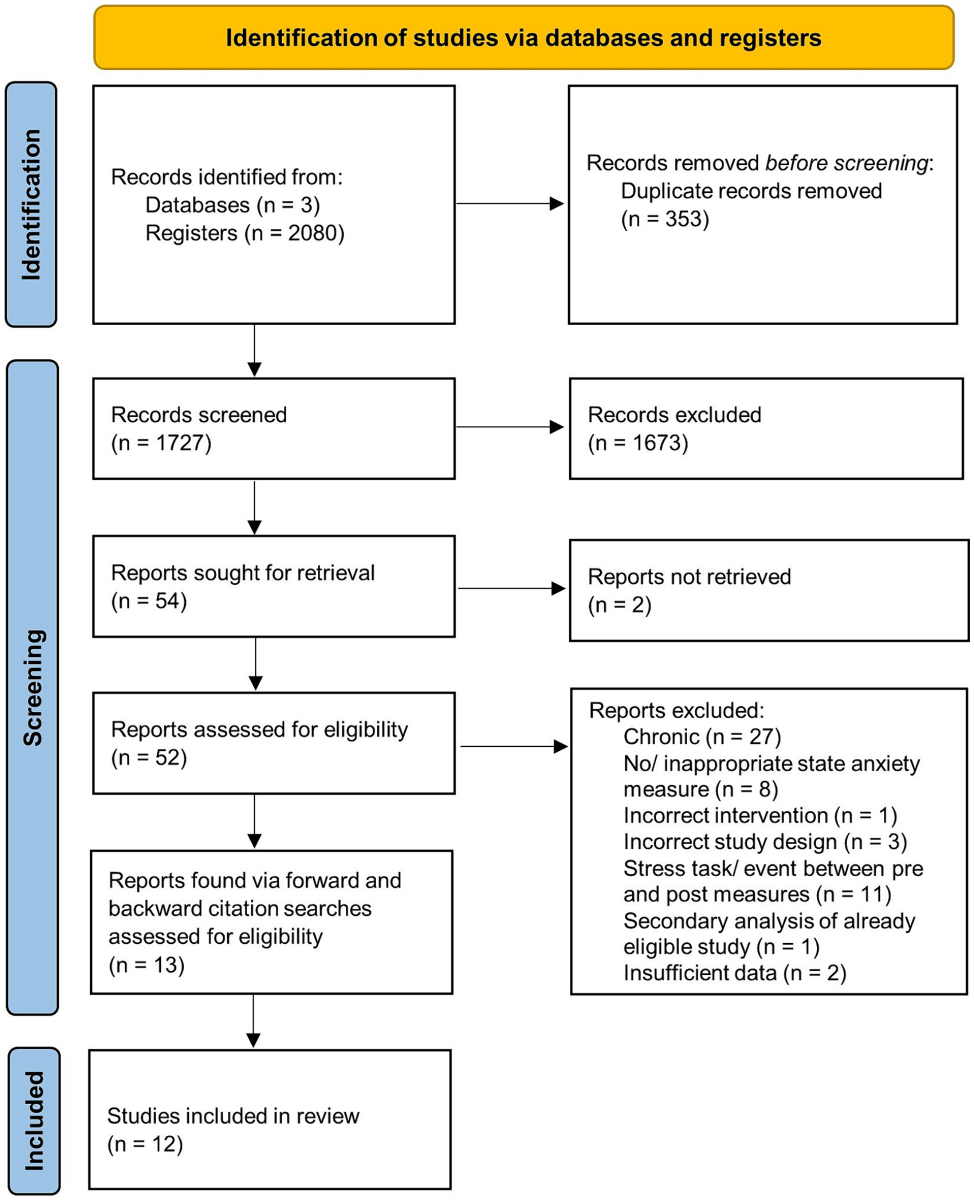
Identification of eligible studies—PRISMA 2020 flow diagram. Note. Source: (Page et al., 2021).

From the 2080 articles identified across three databases, 353 duplicate records were removed. Study titles and abstracts were screened for relevance against the inclusion criteria by PC. Where there was any uncertainty, additional screening was conducted by OH. Studies were most often excluded at this stage due to the population examined (i.e., children, medical diagnoses), type of study (i.e., case study, review, meta-analysis, dissertation), lack of an appropriate state anxiety measure, and lack of relevant interventions. Full text was then sought for 54 of the studies deemed as potentially relevant. Two studies were unable to be retrieved and 52 were further assessed for eligibility. Thirteen studies obtained via forward and backward searches from the 54 studies were also assessed. Note that studies assessing longitudinal interventions were considered during eligibility screening if they had described pre-post measures being collected for the first session. Overall, 12 studies were deemed eligible for review (see Figure 1 for a guideline of the screening process illustrated in a PRISMA 2020 flow diagram). Where available, participant demographics (i.e., age, gender, sample size), information regarding the treatment and comparison conditions (i.e., modality, duration), anxiety measures, and pre- and post-intervention results were obtained from the eligible studies.

### Risk of bias assessment

The Cochrane Collaboration’s Risk-of-Bias tool (RoB 2) for randomized parallel and cross-over trials was utilized to assess the quality ratings for the twelve studies. This tool traditionally includes the assessment of five standard domains of potential bias: (1) randomization process, (2) deviations from intended interventions, (3) missing outcome data, (4) measurement of the outcome, and (5) selection of the reported result. For the cross-over trial assessment there was an additional bias from period and carryover effects domain. Risk of bias categories were classified as “high risk of bias,” “some concerns of bias,” and “low risk of bias.” Overall low risk of bias indicates that the study was judged to have low risk of bias across *all* domains. Some concerns to the overall risk of bias indicates that the study was judged to have some concerns to at least one domain of bias. Finally, overall high risk of bias indicates that the study was judged to have either a high risk of bias judgement in at least one domain, or judgements of some concern in multiple domains of risk of bias.

### Meta-analyses

Meta-analyses were conducted using Review Manager Web (RevMan) using the more conservative random effect model for continuous data, set to inverse variance and a 95% confidence interval (DerSimonian and Laird, 1986). It assumes the true effect may vary between studies due to heterogeneity (Dettori et al., 2022). Hedge’s adjusted g was calculated using the standardised mean difference (SMD) to assess the size of the acute effects of each intervention. Hedge’s *g* is the equivalent of the Cohen’s *d*, but with the adjustment for small sample bias and standard error. Hedge’s *g* was calculated using the mean difference between pre- and post-intervention values, and the standard deviation of the mean difference (SD_diff_) for both intervention and control group. These values were then input into RevMan to compute the Hedge’s *g* between the intervention and control groups. The standard deviation of the mean difference (SD_diff_) is a data value commonly missing in studies, hence a similar approach to the methodology by Yagiz et al. (2021) was taken to produce estimations of the SD_diff_. Most studies lacked the required information to calculate validated approximations based on available data (i.e., means, standard deviations, confidence intervals, *p*-values, *t*-values, *F*-values and standard errors) via RevMan. Subsequently, the corresponding authors were contacted via email to request their full datasets or alternatively the mean difference and SD_diff_ if available. Only two studies were able to provide their full data sets within the required time frame (Balban et al., 2023; Wolfe et al., 2023). Therefore, for all studies, the SD_diff_ value was calculated using the formula below, provided by the Cochrane Handbook for Systematic Reviews of Interventions (Higgins et al., 2023):


$${SD}_{\mathrm{diff}}=\sqrt{{SD}_{pre}^{2}+{SD}_{\mathrm{post}}^{2}-\left(2\times r\times {SD}_{pre}\times {SD}_{\mathrm{post}}\right)}$$

Note that the SD_diff_ value was calculated by assigning *r* in the formula the value of 0.7 (as recommended) to provide a conservative estimate of the effect size (Rosenthal, 1993; Higgins et al., 2023). This approach has been executed by several previous systematic reviews and meta-analyses (Papadopoulos et al., 2020; Yagiz et al., 2021). For methodological consistency, we applied the formula above to calculate the SD_diff_ for all included studies (see Supplementary Table S2 for a summary of the calculated effect sizes).

The rule-of-thumb interpretation of Hedge’s *g* follows the same convention as for Cohen’s *d*, i.e., small (0.2), medium (0.5), or large (0.8) (Cohen, 1988). A negative effect size score indicates favor towards to treatment condition, whereas a positive effect size score indicates favor towards the comparison condition.

To examine the heterogeneity of the data, we utilized the chi-squared (*χ*^2^) and *I*^2^ statistics. The *χ*^2^ statistic and the associated *p*-value indicate whether the true effect sizes of the studies were similar or significantly different. A low *p*-value and large *χ*^2^ statistic relative to the degrees of freedom indicate evidence of heterogeneity of intervention effects (Higgins et al., 2023). The *I*^2^ statistic indicates the percentage of variation in effect estimates across studies attributed to heterogeneity rather than chance. It is interpreted as low (25%), moderate (50%) and high (75%) (Higgins et al., 2003). A high percentage indicates a greater amount of variation between effect sizes, due to differences in factors such as study design, sample demographics, and methodological discrepancies. Analyses were split into four categories based on the interventions present in twelve studies: (1) cognitive-based interventions versus control; (2) embodiment practices versus control; (3) breathing-based interventions versus control; and (4) combined interventions versus control. Note that we also conducted additional analysis to directly compare the relationship between breathing-based interventions versus combined interventions with the available data (see Supplementary material).

### Publication bias consideration

We considered the influence of publication bias by examining the funnel plot (Supplementary Figure S3) and performing an Egger’s test across all included studies (Egger et al., 1997; Duval and Tweedie, 2000). This method assumes that should no publication bias be present, the effect sizes of the studies will be equally dispersed on either side of the overall effect. Otherwise, should publication bias be present, the funnel plot will appear asymmetric (Duval and Tweedie, 2000). The Egger’s test, a linear regression test, can be used in the latter case to provide more objective evidence to support the presence of publication bias (*p* < 0.05) (Egger et al., 1997). Due to the limited number of studies in each analysis, and the literature recommends at least 10 studies to test for funnel plot asymmetry (Higgins et al., 2023), we decided to measure publication bias using the studies included across *all* meta-analyses.

## Results

Twelve study reports were eligible for inclusion in the meta-analysis (Pawlow and Jones, 2002, 2005; Khemka et al., 2009; Zeidan et al., 2010; Telles et al., 2012; McWhorter and Gil-Rivas, 2014; Lancaster et al., 2016; Telles et al., 2019; Bellosta-Batalla et al., 2020; Balban et al., 2023; Nien et al., 2023; Wolfe et al., 2023). In accordance with our inclusion criteria, all of these studies were randomized controlled trials; additionally, three of these studies had a cross-over design (Telles et al., 2019; Nien et al., 2023; Wolfe et al., 2023). Further information about participant demographics, treatment and comparison conditions, anxiety measures, and pre- and post-intervention results are provided in the corresponding tables in the meta-analyses section under each relevant analysis. Note that the results for the meta-analysis of studies directly comparing the effects of breathing-based intervention against combined modality interventions (with no control condition) have been included in Supplementary material.

### Risk of bias

The five sections of the Cochrane Collaboration’s risk of bias tool 2 (RoB 2) using the Excel tool provided by the risk of bias website supported by Cochrane (Sterne et al., 2019) were used to assess the risk of bias in the 9 randomized parallel trials and 3 cross-over trials. Overall, three studies were judged to have low overall risk of bias (McWhorter and Gil-Rivas, 2014; Lancaster et al., 2016; Bellosta-Batalla et al., 2020), eight studies were judged to have some concern regarding overall risk of bias (Pawlow and Jones, 2002, 2005; Khemka et al., 2009; Telles et al., 2012, 2019; Balban et al., 2023; Nien et al., 2023; Wolfe et al., 2023), and one study was judged to have high overall risk of bias (Zeidan et al., 2010). It should be considered that given the nature of behavioral intervention studies, which typically requires an interventionist to administer instructions or play the audio-clip, the assessment of blinding of participants and personnel was omitted which may have inflated the risk of bias judgements. Figures provided in the Supplementary material present the risk of bias summary and graphs of the author’s conclusions about each domain for the eligible randomized controlled parallel trials and cross-over design trials.

### Publication bias consideration

Results from the funnel plot inspection showed a significant outlier which likely influenced the overall effect size (Pawlow and Jones, 2005) (Supplementary Figure S3). The observed funnel plot asymmetry due to this outlier suggests presence of publication bias (available in Supplementary material), supported by Egger’s regression test (intercept = −9.28; *t* = 4.58; *p* < 0.001). To correct for this, the outlier study conducted by Pawlow and Jones (2005) was excluded from the embodiment-based intervention versus control meta-analysis (unadjusted analysis available in Supplementary material). Removing this study from the analysis resulted in a more symmetrical funnel plot, with further Egger’s regression test suggesting reduced presence of publication bias (intercept = −5.24; *t* = 2.09; *p* = 0.06). Note that potential alternative explanations for the initial asymmetry observed in the funnel plot could be due to differences in methodology quality and true heterogeneity (Higgins et al., 2023). Overall, this highlights the need to further develop the body of literature targeted towards brief intervention for anxiety, regardless of positive or negative outcomes to reduce the risk of publication bias in the future.

### Meta-analyses

#### Cognitive-based intervention versus control

**Figure 2:**
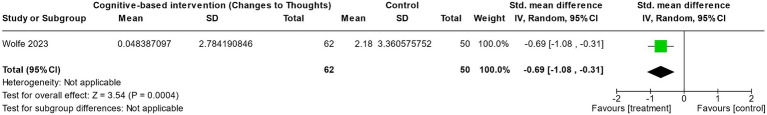
The effect of an acute cognitive reappraisal therapy (involving active change to thoughts) on reducing state anxiety compared to a control group.

A single brief cognitive-based intervention, which focused on actively changing one’s thoughts using a cognitive reappraisal strategy, had a moderate effect size in reducing state anxiety when compared to a control (*g* = −0.69; confidence interval (CI) [−1.08; −0.31]; Figure 2 and Table 1). This was a significant effect (*Z* = 3.54; *p* = 0.0004). Note that there were no studies focusing on brief interventions involving only attention towards one’s thoughts (rather than active changes) that were eligible to be included in this analysis.

**Table 1:** Characteristics of eligible acute cognitive-based intervention studies.

Author(s), Date	Population	Anxiety Measure	Interventions	*N*	Mean (SD) Pre-intervention score	Mean (SD) Post-intervention score
Wolfe et al., 2023	Adults 58.6% F, 51.4% M Aged 18–35 years	STICSA-S	Cognitive reappraisal 16.5 min Audio recording	62	13.92 (2.83)	13.97 (3.00)
			Basic instruction unrelated to thoughts and emotions (Attention control) 11.7 min Audio recording	50	14.64 (3.31)	16.82 (4.23)

STICSA-S, State-Trait Inventory of Cognitive and Somatic Anxiety-State Somatic Version.

#### Embodiment interventions versus control

**Figure 3:**
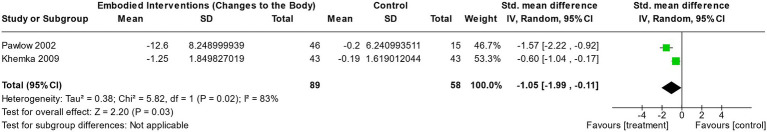
The effect of acute embodiment interventions (involving active change to the body) on reducing state anxiety compared to a control group. Outlier removed to adjust for publication bias.

Acute administration of embodiment interventions (specifically interventions that involve active changes to one’s body/muscles) significantly reduced state anxiety compared to control conditions (*g* = −1.05; CI [−1.99; −0.37]; Figure 3 and Table 2). In particular, progressive muscle relaxation (Pawlow and Jones, 2002) showed a large effect in reducing state anxiety (*g* = −1.57; CI [−2.22; −0.92]; although the two studies show a similar trend in favor of the embodiment interventions, heterogeneity was high (*I*^2^ = 83%; *χ*^2^ = 5.82; *p* = 0.02). Note that the studies involved embodiment interventions focusing on the purposeful changes to one’s body. Similar to that of the cognitive-based interventions above, no studies focusing solely on attention towards one’s body were found that were eligible to be included for analysis.

**Table 2:** Characteristics of eligible acute embodiment intervention studies.

Author(s), Date	Population	Anxiety Measure	Interventions	*N*	Mean (SD) Pre-intervention Score	Mean (SD) Post-intervention Score
Pawlow and Jones, 2002	Undergraduate students 30F, 31 M Mean age 23.23 [4.78] years	STAI	PMR 20–25 min Live training*	46	38 (10.9)	25.4 (4.9)
			Quiet sitting 30 min	15	35.6 (7.4)	35.3 (8.5)
Pawlow and Jones, 2005	Undergraduate students 29F, 26 M Mean age 23.96 [7.54] years	STAI	PMR 20–25 min Live training*	41	33.05 (1.43)	25.17 (1.25)
			Quiet sitting 25 min	14	37.21 (2.44)	36.57 (2.13)
Khemka et al., 2009	Volunteers 30F, 56 M Mean age 30.14 years (DRT group) 28.35 (Supine rest group)	SAS	Deep relaxation technique (guided relaxation of tense areas) 20 min Audio recording	43	8.3 (2.48)	7.05 (2.27)
			Supine rest 20 min	43	7.98 (2.1)	7.79 (2.08)

STAI, Spielberger State-Trait Anxiety Inventory-State Version; SAS, State-Trait Anxiety Inventory A-State; PMR, progressive muscle relaxation. *Live-training-facilitator/experimenter present in session to teach and demonstrate technique to participants.

#### Breathing-based intervention versus control

**Figure 4:**
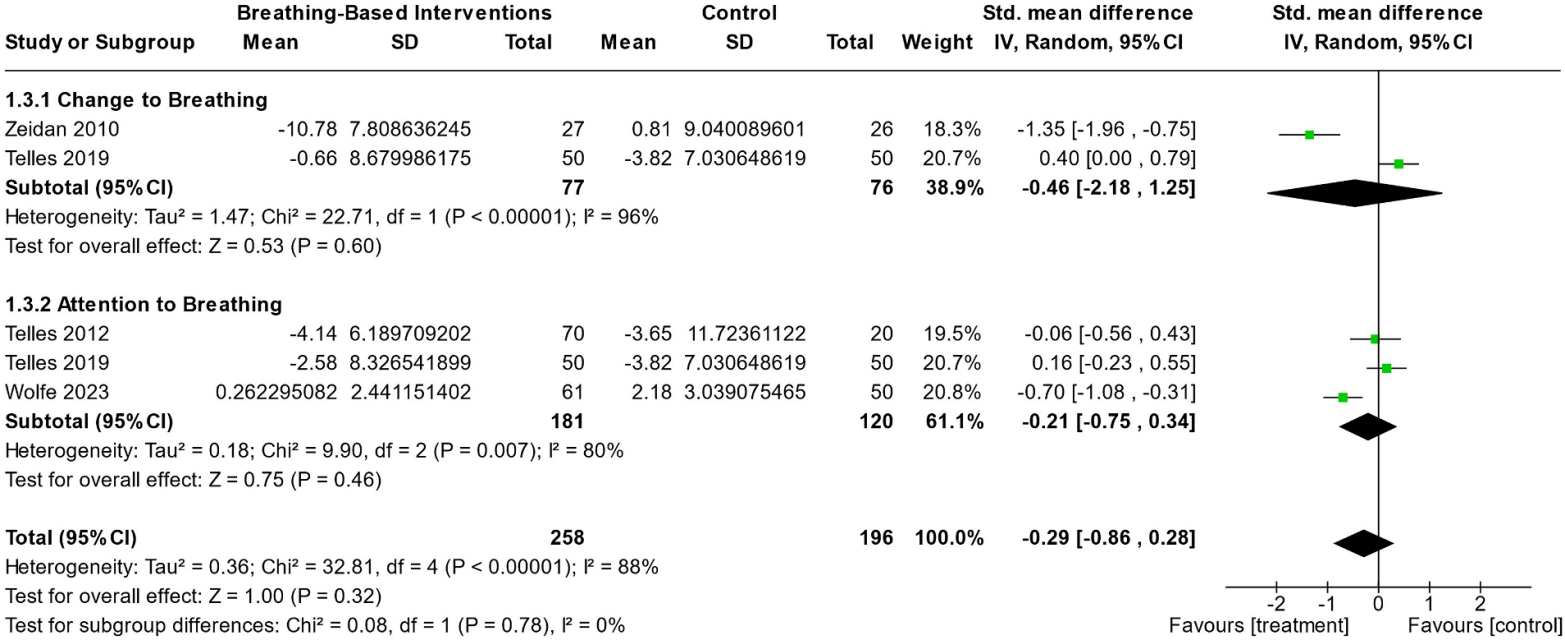
The effect of acute breath-based interventions (both active changes to breathing and passive attention towards breathing) on reducing state anxiety compared to a control group.

Overall, interventions focused on breathing did not produce a significant reduction in state anxiety when compared to control conditions (Figure 4 and Table 3). While interventions that involved the manipulation and conscious change to breathing showed a slightly greater effect size favoring the breath-based intervention over control (*g* = −0.46; CI [−2.18; 1.25]) compared to interventions that mainly focused on one’s attention to breathing (*g* = −0.21; CI [−0.75; 0.34]), neither were significant. Furthermore, the larger effect within conscious changes to breathing was driven by the large effect found in Zeidan et al. (2010), who utilized 20 min of deep breathing exercises (*g* = −1.35; CI [−1.96; −0.75]), while alternate nostril breathing employed by Telles et al. (2019) produced significantly smaller reductions in state anxiety than the control condition (*g* = 0.40; CI [0.00; 0.79]). Note that both types of breathing interventions indicated high heterogeneity, as displayed in the relatively wide confidence interval (Change to breathing *I*^2^ = 96%; *χ*^2^ = 22.71; *p* < 0.00001; Attention to breathing *I*^2^ = 80%; *χ*^2^ = 9.9; *p* = 0.007).

**Table 3:** Characteristics of eligible breathing-based intervention studies.

Author(s), Date	Population	Anxiety Measure	Interventions	*N*	Mean (SD) Pre-intervention Score	Mean (SD) Post-intervention Score
Change to Breathing Interventions versus Control						
Zeidan et al., 2010	Undergraduate students 48F, 34 M Median age 19 years	STAI	Deep breathing exercises 20 min Live training*	27	40.19 (10.80)	29.41 (6.34)
			Sitting 20 min	26	35.19 (10.47)	36.0 (12.41)
Telles et al., 2019	Healthy male volunteers Mean age 28.4 [8.2] years	STAI	Alternate nostril breathing (ANB) 18 min	50	38.66 (10.94)	38.00 (11.44)
			Quiet sitting 18 min	50	38.06 (8.83)	34.24 (9.29)
Attention to Breathing Interventions versus Control						
Telles et al., 2012**	Indian army males and healthy males Mean age 33.7 [7.0] years	STAI	Breath awareness 45 min Live training*	70	43.71 (9.09)	44.21 (8.50)
			Music 45 min Audio recording	20	44.90 (15.16)	41.25 (15.11)
Telles et al., 2019	Healthy male volunteers Mean age 28.4 [8.2] years	STAI	Breath awareness (BAW) 18 min	50	37.70 (10.93)	35.12 (10.55)
			Quiet sitting 18 min	50	38.06 (8.83)	34.24 (9.29)
Wolfe et al., 2023	Adults 58.6% F, 51.4% M Aged 18–35 years	STICSA-S	Breath awareness 17.5 min Audio recording	61	14.25 (2.71)	14.51 (3.38)
			Basic instruction unrelated to thoughts and emotions (Attention control) 11.7 min Audio-recording	50	14.64 (3.31)	16.82 (4.23)

STAI, Spielberger State-Trait Anxiety Inventory-State Version; STICSA-S, State-Trait Inventory of Cognitive and Somatic Anxiety-State Somatic Version. *Live-training-facilitator/experimenter present in session to instruct, teach and demonstrate technique to participants. **Telles et al. (2012) included three intervention groups: yoga, breath awareness, and music control. We included data from the breath awareness and the control group as this was more relevant to the research criteria.

#### Combined modality interventions versus control

**Figure 5:**
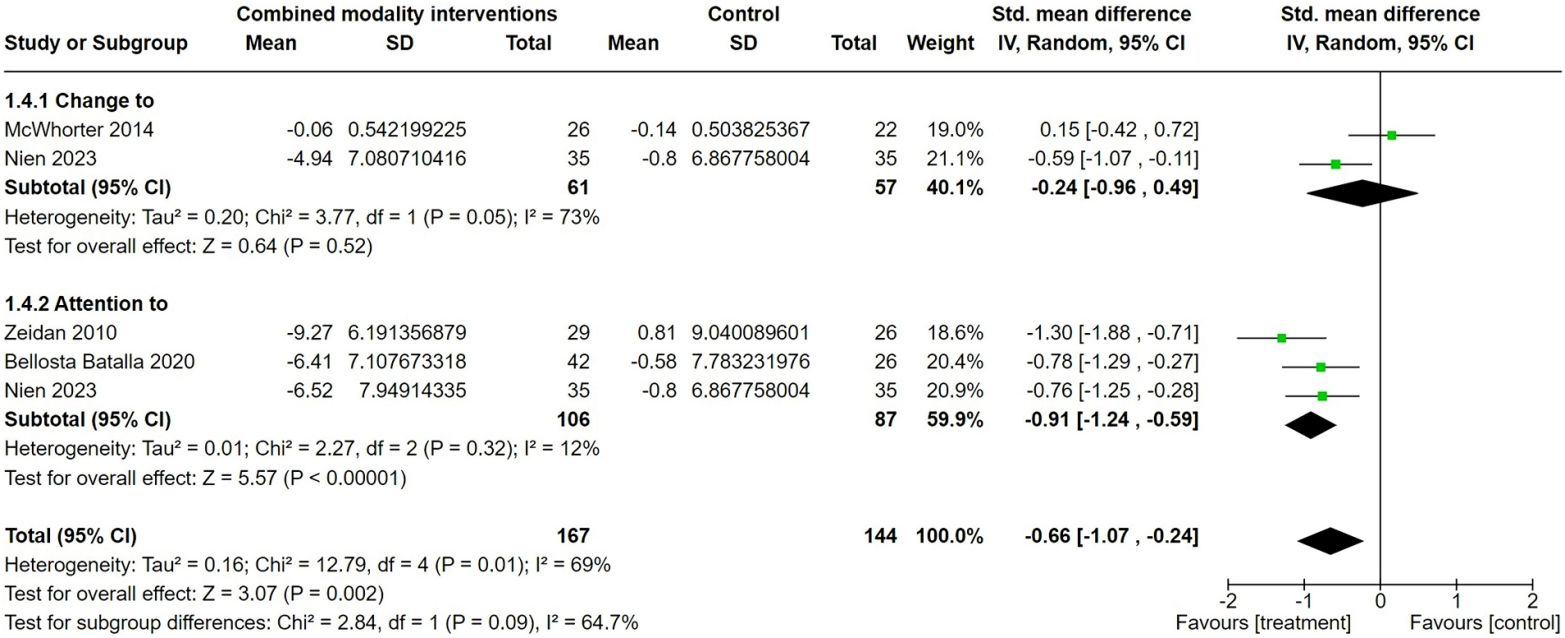
The effect of acute therapies that combined breathing, embodiment and/or cognitive-based interventions (both with active changes or passive attention) on reducing state anxiety when compared to a control group.

Finally, the combination of either breathing, body and/or cognitive practices (similar to the approaches taken in more recent third-wave therapies) produced a significant reduction in state anxiety compared to control conditions (*g* = −0.66; CI [−1.07; −0.24]; Figure 5 and Table 4). Unlike with isolated breathing-based interventions, studies that involved interventions that focused on attention towards the breath and body (Bellosta-Batalla et al., 2020), attention towards the breath and cognition (Zeidan et al., 2010) and attention towards breath, body and cognition (Nien et al., 2023) had a greater effect size (*g* = −0.91; CI [−1.24; −0.59]) when compared to interventions that focused on changes to one’s breathing and body (*g* = −0.24; CI [−0.96; 0.49]) (McWhorter and Gil-Rivas, 2014; Nien et al., 2023). The difference between these two sub-groups trended towards significance (*χ*^2^ = 2.84; *I*^2^ = 64.7%; *p* = 0.09). Heterogeneity was low in the attention-to combined modalities sub-group (*I*^2^ = 12%; *χ*^2^ = 2.27; *p* = 0.32) and moderate in the change-to combined modalities sub-group (*I*^2^ = 73%; *χ*^2^ = 3.77; *p* = 0.05).

**Table 4:** Characteristics of eligible combined modality intervention studies.

Author(s), Date	Population	Anxiety Measure	Interventions	*N*	Mean (SD) Pre-intervention Score	Mean (SD) Post-intervention Score
Change to Combined Modality Interventions versus Control						
McWhorter and Gil-Rivas, 2014	Undergraduate students 32F, 16 M Mean age 23.53 [8.39] years	STAI 4-point Likert scale	Functional relaxation (Directed body relaxation upon exhalation) 15 min Audio recording	26	1.52 (0.66)	1.46 (0.73)
			Quiet sitting 15 min	22	1.70 (0.66)	1.56 (0.64)
Nien et al., 2023	Track and field athletes 8F, 27 M Mean age 20.63 [2.43] years	STAI-Chinese version	Relaxation (Change to breath and body) 30 min Audio recording	35	36.94 (9.51)	32.00 (8.66)
			Quiet sitting 30 min	35	34.86 (8.03)	34.06 (9.40)
Attention to Combined Modality Interventions versus Control						
Zeidan et al., 2010	Undergraduate students 48F, 34 M Median age 19 years	STAI	Mindfulness (Attention to breathing and cognition) 20 min Live training*	29	40.93 (8.47)	31.66 (7.25)
			Sitting 20 min	26	35.19 (10.47)	36.0 (12.41)
Bellosta-Batalla et al., 2020	Undergraduate and postgraduate students 51F, 17 M Mean age 24.0 [5.0] years	STAI-Spanish version	Brief mindfulness (attention to breath and body) 90 min Facilitator-led	42	17.24 (9.86)	10.83 (7.87)
			Empathy and creativity exercise 90 min	26	16.23 (9.94)	15.65 (10.15)
Nien et al., 2023	Track and field athletes 8F, 27 M Mean age 20.63 [2.43] years	STAI-Chinese version	Mindfulness (attention to breath, body and cognition) 30 min Audio recording	35	37.83 (10.82)	31.31 (9.44)
			Quiet sitting 30 min	35	34.86 (8.03)	34.06 (9.40)

STAI, Spielberger State-Trait Anxiety Inventory-State Version; STICSA-S, State-Trait Inventory of Cognitive and Somatic Anxiety-State Somatic Version. *Live-training-facilitator/experimenter present in session to instruct, teach and demonstrate technique to participants.

## Discussion

### Main findings

This review aimed to provide an overview of the effects of acute administration of cognitive, embodiment, breathing, or combined modality interventions commonly used to reduce state anxiety in healthy populations. It also aimed to discern, in cases where such data was available, whether more active approaches (i.e., changes to) or passive approaches (i.e., attention towards) of such modalities would present differences in intervention effectiveness. Overall, the available literature indicated that cognitive, embodiment and combination therapies significantly reduced state anxiety, while breathing interventions produced mixed results. Available cognitive and embodiment practices only covered active approaches, and thus could not be compared to passive attention-only strategies. Breathing and combination interventions were the only classes to cover both active changes and passive attention approaches, with both breathing sub-groups producing mixed results. However, passive attention to combined modalities produced a significant (and significantly greater) reduction in state anxiety when compared to active change strategies. Therefore, interoceptive foci can be successfully utilized to reduce state anxiety, although these are not necessarily imperative for therapeutic success. Finally, heterogeneity across studies was deemed to be high for the majority of analyses, which should be considered upon further detailed interpretation of results below.

#### Cognitive-based intervention versus control

Only one study meeting all inclusion criteria utilized a solely cognitive-based intervention for state anxiety (Wolfe et al., 2023). In this intervention, a 16.5 min session of cognitive reappraisal showed a moderate effect size in reducing state anxiety compared to an 11.7 min control session. This technique utilized an active change in one’s thought processes, where participants received audio instructions to reframe negative emotions and perceive a stressful situation with more positive explanations and implications. Comparatively, no available studies took a passive attention-only approach to cognition. Therefore, while promising in nature, the limited literature hinders the ability to draw firm conclusions regarding the efficacy of brief cognitive-based strategies to reduce state anxiety.

#### Embodiment interventions versus control

The available literature demonstrated evidence supporting the use of purposeful and directed changes (i.e., relaxation) to one’s body to reduce state anxiety in healthy adults. Both progressive muscle relaxation, which involves the purposeful contraction and relaxation of set muscle groups in the body (Pawlow and Jones, 2002) and deep relaxation training, which involves a guided relaxation of tense areas of the body (Khemka et al., 2009), reduced state anxiety with large and moderate effect sizes (respectively). It has been hypothesized that these techniques may act through interoceptive mechanisms, where attention is brought towards the body to improve an individual’s interoceptive ability, allowing for the amelioration of anxiety (Weng et al., 2021). As no studies were available that considered only attention towards the body, it is unclear whether attention alone is enough to initiate these effects, or whether purposeful relaxation and likely reduction in sympathetic tone (Conrad and Roth, 2007) is required for the reduction in state anxiety to occur.

#### Breathing-based interventions versus control

Interestingly, breathing-based interventions demonstrated mixed effects on state anxiety. These inconsistencies further persisted upon separating analyses into sub-groups of active (i.e., changes to) and passive (i.e., attention towards) breathing. Importantly, the efficacy of active breathing interventions, which encourage purposeful changes to breathing, appears to depend heavily on the type of breath-work technique used. For instance, 20 min of deep breathing exercises was shown to have a large, significant effect in reducing state anxiety compared to a control condition (Zeidan et al., 2010). While this may reduce sympathetic tone and thus state anxiety (Zaccaro et al., 2018), modulating descending and ascending interoceptive signaling may also play an important anxiolytic role (Weng et al., 2021). Further investigation into the physiological, perceptual and psychological mechanisms at play in this technique would be a helpful addition to this literature. Additionally, considering how previous literature has indicated that deep breathing in some cases can worsen anxiety (Meuret and Ritz, 2010; Maleki et al., 2022), it would also be beneficial to determine such mechanisms to determine which pathways hyperventilation and/or hypocapnic breathing may also enact upon to exacerbate anxiety.

Comparatively, an 18 min session of alternate nostril breathing (ANB) was unable to produce effective reductions in state anxiety, with the control group (quiet sitting) experiencing a significantly larger anxiolytic effect (Telles et al., 2019). While it is hypothesized that alternate nostril breathing may be too difficult for individuals to master in the short term due to difficulties with reduced airflow (Telles et al., 2019; Epe et al., 2021), this result demonstrates that not all active changes in breathing are certain to reduce state anxiety, and caution must be taken when initiating these exercises. However, it is possible that long-term practice for ANB may produce reliable reductions in anxiety, even if the acute effect is not significantly anxiolytic. More passive breath awareness techniques did not produce convincing evidence regarding a reduction in state anxiety. While one study moderately favored breath awareness over a control (Wolfe et al., 2023), mean state anxiety did not significantly decrease with either condition. Two further studies were unable to produce significantly greater effects using breath awareness techniques compared to control conditions of quiet sitting or listening to music (Telles et al., 2012, 2019). Importantly, these results demonstrate that directing attention towards interoceptive breathing sensations alone may not be enough to initiate changes in state anxiety. However, consideration should also be taken for the population of individuals employed for these studies. Both Telles et al. (2012) and Telles et al. (2019) were conducted in a solely Indian male soldier population, and these studies demonstrated weaker effects in both active and passive breathing techniques. It is possible that breathing techniques may not be effective on this population specifically, or confounds existed in the methodology employed for delivery. Finally, it may also be beneficial for future reviews to address the potential for sex differences influencing the effect sizes observed more generally.

#### Combined modality interventions versus control

Interventions utilizing attention towards a combination of modalities are reflective of more recent and widely adopted practices such as mindfulness. Importantly, these mindful practices (including attention to breathing and cognition, attention to breathing and body, and attention to breathing, body and cognition) were shown to have a strong and consistent effect in reducing state anxiety when compared to a control (Zeidan et al., 2010; Bellosta-Batalla et al., 2020; Nien et al., 2023). Furthermore, heterogeneity between studies was markedly low, suggesting that numerous interventions involving mindfulness-based strategies may be beneficial for altering anxiety. It is possible that these combination strategies may be simultaneously enhancing both interoceptive and cognitive pathways for improved efficacy.

However, not all therapies involving active changes to combined modalities have been successful at reducing state anxiety. Specifically, while a 30 min session of relaxation therapy involving active changes to the breath and body moderately reduced state anxiety (Nien et al., 2023), a 15 min session of functional relaxation (i.e., directed body relaxation upon exhalation) favoured the quiet sitting control (McWhorter and Gil-Rivas, 2014). Therefore, while passive mindfulness practices produce convincing reductions in state anxiety, interventions that actively evoke changes in cognition, the body or breathing may need to be approached with more caution.

### Limitations

There are several limitations to consider when interpreting the current findings. Firstly, although a substantial number of observational studies exist, there were a limited number of randomized controlled trial studies that assessed acute psychotherapeutic interventions on state anxiety that could be included in our meta-analyses. This likely contributed to the high levels of heterogeneity reported, and individual studies thus had a greater influence on the direction and strength of the overall effect size. Further research into the acute effects of cognitive, body, breath-based, and combined modality interventions will lead to stronger conclusions regarding their effectiveness for reducing state anxiety. Furthermore, while interoceptive links are plausible candidates for mediating therapeutic effects, most studies have not directly measured any resulting changes in interoception alongside anxiety. Therefore, we are currently unable to confirm whether interoceptive changes may have played a moderating role on corresponding effect sizes for anxiety.

Additionally, the risk of bias analysis indicated some issues with randomization, and publication bias is likely to have influenced these results. While studies indicated randomized allocation of participants, one study failed to explicitly indicate randomization (Khemka et al., 2009), four failed to explicitly describe what methods were used to do so (i.e., randomization matrix, block randomization) (Pawlow and Jones, 2002, 2005; Telles et al., 2012; Lancaster et al., 2016), and one randomly allocated participants into groups based on the week of assessment—a strategy not typically endorsed (Zeidan et al., 2010). More transparency regarding randomization, specifically providing explicit details about the randomization method used and the data analysis plan is encouraged. While blinding was also an area of concern, it should be noted that due to the behavioral nature of the interventions investigated, it is difficult to blind both participants and experimenters. The influence of publication bias is also noteworthy. While we have chosen a sensitivity-based correction by excluding outlier studies, further correction techniques could be employed to adjust the reported effect sizes. However, we have chosen to perform comparative sub-group meta-analyses that are available within the RevMan software, which has limited publication bias correction options. Future analyses may wish to consider multiple publication bias correction analyses to further validate the current findings.

Notably, most studies included in the analysis did not report the standard deviation of the mean difference (SD_diff_) for the intervention and comparison groups. As a result, the SD_diff_ was calculated using the equation recommended by Cochrane (Higgins et al., 2023), adding a layer of uncertainty into the effect size estimates. Full reporting of both the mean difference and standard deviation of the mean difference in future studies would help to minimize this effect.

A final limitation of the current summary analysis approach is that this cannot consider how the quality of the therapeutic relationship may have influenced the level of success of certain treatment modalities. As the included interventions varied in their administration methods (i.e., audio-recording versus facilitator-led/live training), it would be interesting to determine whether a more interpersonal connection—i.e. having a facilitator/therapist administering instructions—would moderate the anxiolytic effect. The current analysis also did not consider the variance in the length of interventions (i.e., 11.7–45 min) on the outcomes recorded. A longer intervention may provide a greater opportunity to practice the intervention and perhaps increase the chance to experience an associated anxiolytic effect. When more data are available, it would be beneficial for future research to consider whether both the opportunity to develop a therapeutic relationship and the duration of an intervention could act as moderating factors affecting the reduction in state anxiety via cognitive, embodiment, breathing and/or combined modality interventions.

## Conclusion

Overall, brief administration of single-modality interventions that involve active changes to cognition, body state and breathing can all be effective in reducing state anxiety. In particular, interventions that focus on changes to the body, such as progressive muscle relaxation and deep relaxation training, are consistently effective in reducing state anxiety. Brief interventions of cognitive reappraisal or deep breathing exercises may also be beneficial for state anxiety, but more evidence is needed to support this. Specifically, care must be taken when selecting appropriate breathing exercises to utilize, as not all produce significant anxiolytic effects in an acute setting. Alternatively, when considering the use of combined modality interventions, taking a more passive approach by directing attention towards cognition, body and/or breathing may be more effective than active change approaches for reducing anxiety. This supports the notion that third-wave mindfulness and acceptance-based therapies produce a consistent positive impact on state anxiety. These findings provide initial evidence indicating which interventions may be effective in reducing state anxiety when administered acutely in a single session. Knowledge of the most effective brief strategies to alleviate anxiety may help to direct the development of further preventative tools, and potentially even population-wide promotion of self-help skills. However, limited eligible studies and publication bias calls for more extensive research in this field to allow for reliable conclusions to be drawn. Additionally, while it is likely that many of these interventions utilize interoceptive pathways to contribute to their anxiolytic effects (Cougle et al., 2020; Weng et al., 2021; De Lima-Araujo et al., 2022), this does not appear to be imperative and requires more direct investigation. Further research into the moderating effect of interoceptive pathways on anxiety may have implications for the development of future treatment protocols for subclinical and clinical anxiety in an acute setting.

## Data availability statement

The original contributions presented in the study are included in the article/Supplementary material, further inquiries can be directed to the corresponding authors.

## Author contributions

PC: Conceptualization, Data curation, Formal analysis, Investigation, Methodology, Project administration, Visualization, Writing – original draft, Writing – review & editing. FG: Conceptualization, Writing – review & editing. FB: Conceptualization, Writing – review & editing. BR: Supervision, Writing – review & editing. KS: Conceptualization, Writing – review & editing. OH: Conceptualization, Formal analysis, Methodology, Project administration, Resources, Supervision, Writing – review & editing.
