# Supplementary material for: A systematic review of brief respiratory, embodiment, cognitive, and mindfulness interventions to reduce state anxiety
Source: Front Psychol. 2024 Jun 12;15:1412928. doi: 10.3389/fpsyg.2024.1412928 (PMC11203600; doi:10.3389/fpsyg.2024.1412928)
Supplement: Supplementary file 1 [file Table_1.DOCX]

Supplementary Material

A Systematic Review of Brief Respiratory, Embodied, Cognitive and Mindfulness Interventions to Reduce State Anxiety

**Phoebe Chin, Faye Gorman, Fraser Beck, Bruce R. Russell, Klaas E. Stephan & Olivia K. Harrison**

*** Correspondence:** Corresponding Author: chiph927@student.otago.ac.nz

# Supplementary Figures and Tables

## Supplementary Figures

##
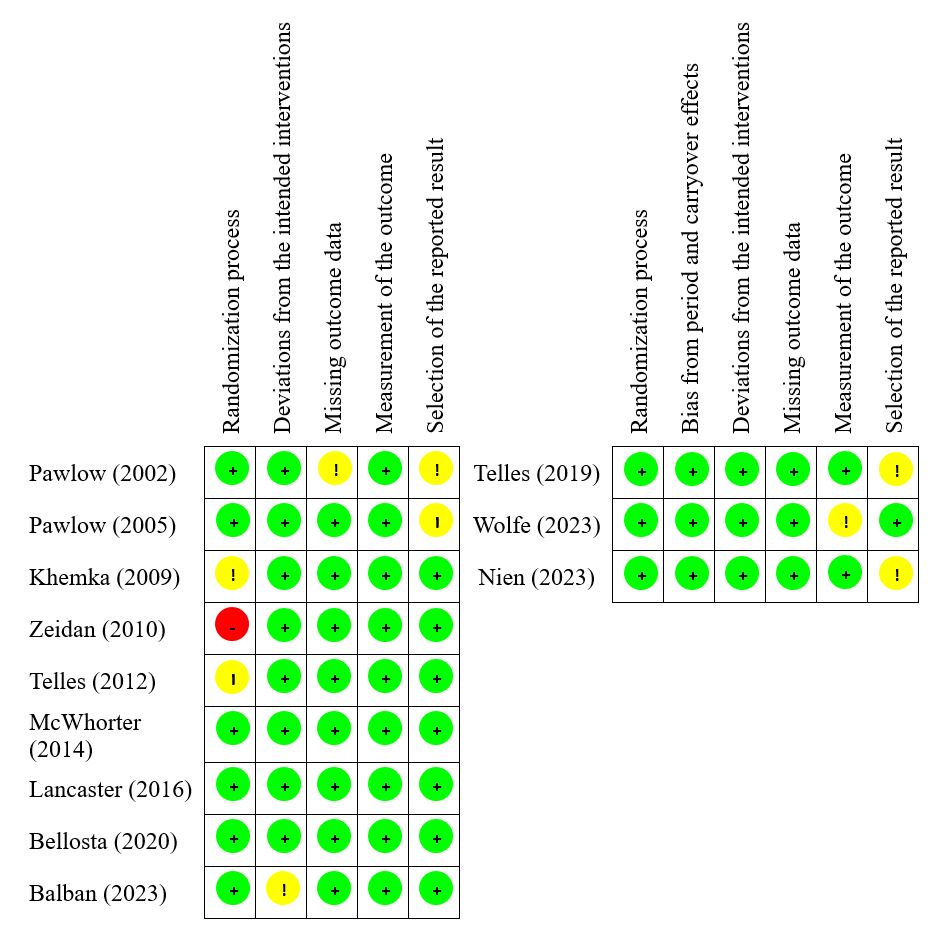


**Supplementary Figure 1.** Judgements for risk of bias domains for each of the eligible randomized controlled trials (left; n = 9) and cross-over design trials (right; n = 3)


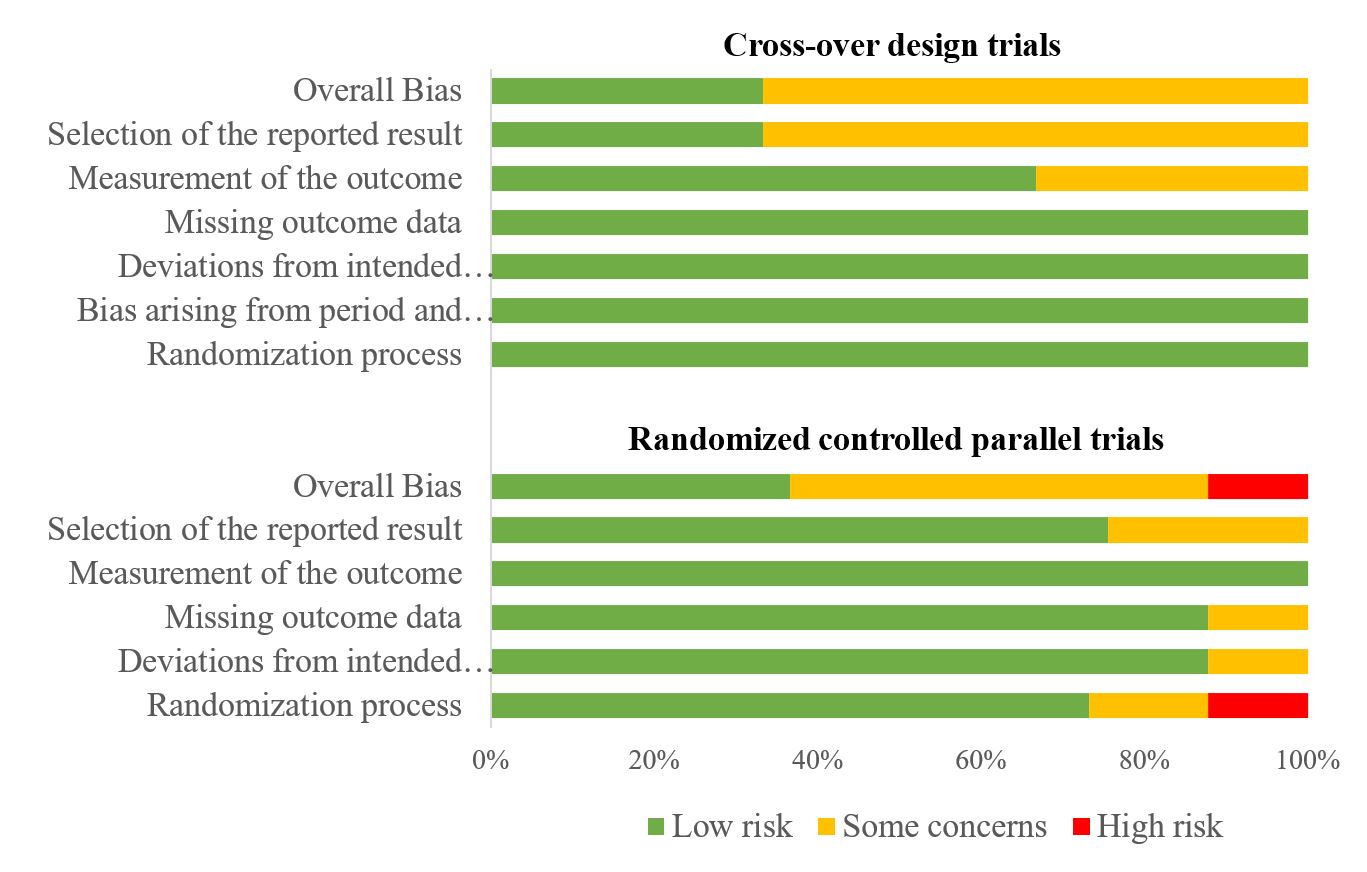


**Supplementary Figure 2.** Percentage of judgements across risk of bias domains for eligible randomized controlled trials (n = 9) and cross-over design trials (n = 3).

**
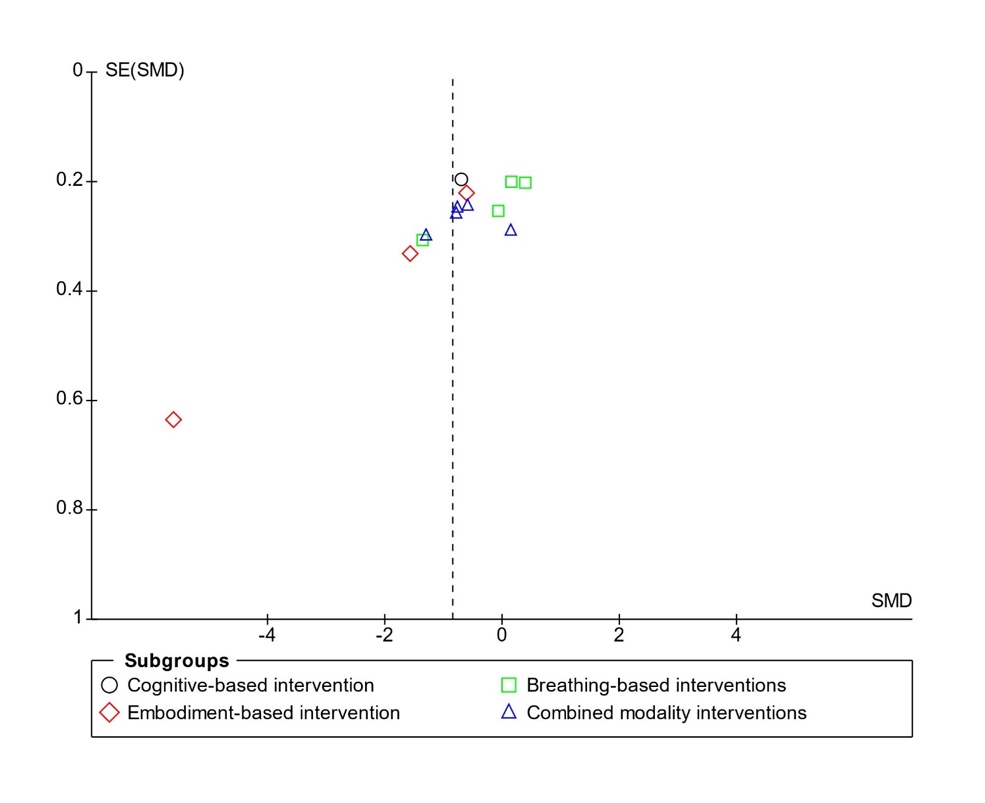
**

**Supplementary Figure 3.** Funnel plot of effect sizes of cognitive, embodiment, breathing-based modality and combined modality interventions in reducing state anxiety, including outlier study. (Black coloured circles represent studies that used cognitive-based intervention, red coloured squares represent studies that used embodiment-based interventions, green coloured squares represent studies that used breathing-based intervention, and blue triangles represent studies that used combined modality interventions. SE(SMD) = standard error of standardised mean differences; SMD = standardised mean difference. Negative SMD represents favor towards the intervention over control.


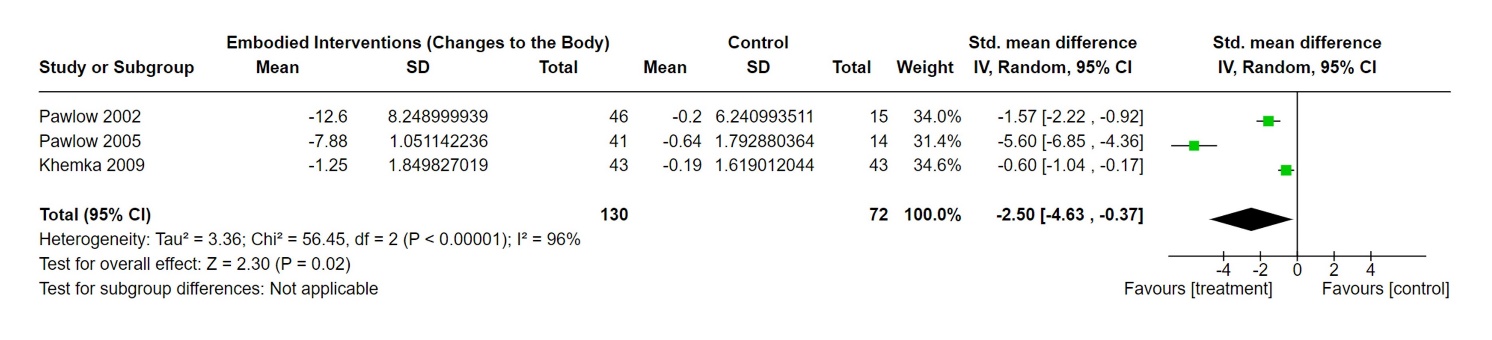


**Supplementary Figure 4.** The effect of acute embodiment interventions (involving active change to the body) on reducing state anxiety compared to a control group. Including outlier study.

**
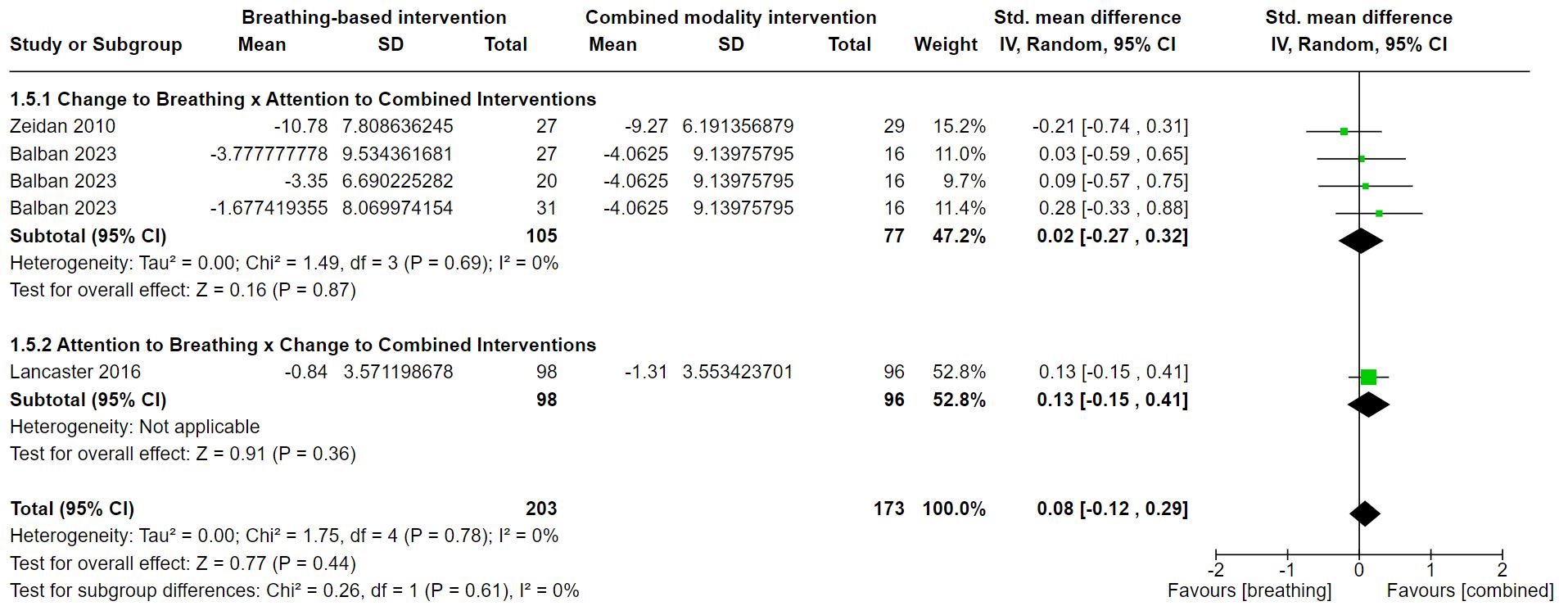
**

**Supplementary Figure 5.** The effect of acute therapies that combined breathing, embodied and/or cognitive-based interventions (both with active changes or passive attention) on reducing state anxiety when compared to breath-based interventions (both active changes to breathing and passive attention towards breathing)

## Supplementary Tables

**Supplementary Table 1.** Characteristics of eligible combined modality intervention studies

| **Author(s), Date** | **Population** | **Anxiety Measure** | **Interventions** | **N** | **Mean (SD) Pre-intervention Score** | **Mean (SD) Post-intervention Score** |
| --- | --- | --- | --- | --- | --- | --- |
| **Change to Breathing versus Attention to Combined Modality Interventions** | | | | | | |
| Zeidan et al., 2010 | Undergraduate students  48F, 34M  Median age 19 years | STAI | Deep breathing exercises  20 minutes  Live training* | 27 | 40.19 (10.80) | 29.41 (6.34) |
|  |  |  | Mindfulness (Attention to breathing and cognition)  20 minutes  Live training* | 29 | 40.93 (8.47) | 31.66 (7.25) |
| Balban et al. (2023) | Adults  M/F unavailable due to data extraction  Mean age 27.97 [13.46] | STAI | Cyclic sighing  5 minutes  Instructional video | 27 | 40.41 (12.82) | 36.63 (11.64) |
|  |  |  | Box breathing  5 minutes  Instructional video | 20 | 34.40 (9.30) | 31.05 (7.32) |
|  |  |  | Cyclic hyperventilation  5 minute  Instructional video | 31 | 36.74 (11.11) | 35.07 (9.24) |
|  |  |  | Mindfulness meditation  5 minutes  Instructional video | 16 | 43.31 (12.67) | 39.25 (10.14) |

Note: STAI – Spielberger State-Trait Anxiety Inventory – State Version

* Live-training – facilitator/ experimenter present in session to instruct, teach and demonstrate technique to participants.

**Supplementary Table 2**. Effect sizes of eligible studies as calculated using Review Manager

| **Author(s), Date** | **Interventions** | **M_diff_** | **SD_diff_** | **Effect size (g)** |
| --- | --- | --- | --- | --- |
| **Cognitive-based Intervention versus Control** | | | | |
| Wolfe et al., 2023 | Cognitive reappraisal | 0.048 | 2.784 | -0.69 [-1.08, -0.31] |
|  | Basic instruction unrelated to thoughts and emotions (Control) | 2.180 | 3.361 |  |
| **Embodiment Intervention versus Control** | | | | |
| Pawlow and Jones, 2002 | Progressive muscle relaxation | -12.600 | 8.249 | -1.57 [-2.22, -0.92] |
|  | Quiet sitting (Control) | -0.200 | 6.241 |  |
| Pawlow and Jones, 2005 | Progressive muscle relaxation | -7.880 | 1.051 | -5.60 [-6.85, -4.36] |
|  | Quiet sitting (Control) | -0.640 | 1.793 |  |
| Khemka et al., 2009 | Deep relaxation technique (guided relaxation of tense areas) | -1.250 | 1.850 | -0.60 [-1.04, -0.17] |
|  | Supine rest (Control) | -0.190 | 1.619 |  |
| **Change to Breathing Intervention versus Control** | | | | |
| Zeidan et al., 2010 | Deep breathing exercises | -10.780 | 7.809 | -1.35 [-1.96, -0.75] |
|  | Sitting (Control) | 0.810 | 9.040 |  |
| Telles et al., 2019 | Alternate nostril breathing | -0.660 | 8.680 | 0.40 [0.00, 0.79] |
|  | Quiet sitting (Control) | -3.820 | 7.031 |  |
| **Attention to Breathing Intervention versus Control** | | | | |
| Telles et al., 2012 | Breath awareness | -4.140 | 6.190 | -0.06 [-0.56, 0.43] |
|  | Music (Control) | -3.650 | 11.723 |  |
| Telles et al., 2019 | Breath awareness | -2.580 | 8.327 | 0.16 [-0.23, 0.55] |
|  | Quiet sitting (Control) | -3.820 | 7.031 |  |
| Wolfe et al., 2023 | Breath awareness | 0.262 | 2.441 | -0.70 [-1.08, -0.31] |
|  | Basic instruction unrelated to thoughts and emotions (Control) | 2.180 | 3.039 |  |
| **Change to Combined Modality Interventions versus Control** | | | | |
| McWhorter and Gil-Rivas, 2014 | Functional relaxation (Directed body relaxation upon exhalation) | -0.060 | 0.542 | 0.15 [-0.42, 0.72] |
|  | Quiet sitting (Control) | -0.140 | 0.504 |  |
| Nien et al., 2023 | Relaxation (Change to breath and body) | -4.940 | 7.081 | -0.59 [-1.07, -0.11] |
|  | Quiet sitting (Control) | -0.800 | 6.868 |  |
| **Attention to Combined Modality Intervention versus Control** | | | | |
| Zeidan et al., 2010 | Mindfulness (Attention to breathing and cognition) | -9.270 | 6.191 | -1.30 [-1.88, -0.71] |
|  | Sitting (Control) | 0.810 | 9.040 |  |
| Bellosta-Batalla et al., 2020 | Brief mindfulness (Attention to breath and body) | -6.410 | 7.108 | -0.78 [-1.29, -0.27] |
|  | Empathy and creativity exercise (Control) | -0.580 | 7.783 |  |
| Nien et al., 2023 | Mindfulness (Attention to breath, body and cognition) | -6.520 | 7.949 | -0.76 [-1.25, -0.28] |
|  | Quiet sitting (Control) | -0.800 | 6.868 |  |

Note: M_diff_ – difference of the means, SD_diff_ – difference of the standard deivations of the mean, g – Hedge’s g
